# Supplementary material for: Observation of the molecular response to light upon photoexcitation
Source: Nat Commun. 2020 May 1;11:2157. doi: 10.1038/s41467-020-15680-4 (PMC7195484; doi:10.1038/s41467-020-15680-4)
Supplement: Supplementary file 1 — Supplementary Information [file 41467_2020_15680_MOESM1_ESM.pdf]

Supplementary Information

Observation of the molecular response to light upon photoexcitation

Yong *et al.*

Supplementary Note 1. Decomposition of Isotropic and Anisotropic Signal

The anisotropic and isotropic scattering signals were separated using a well-established method[1]. The decomposition equation is given by:

$$\Delta I(\phi, q) = \frac{1}{2}\left(3\left(\cos(\phi)\sqrt{1-\left(\frac{\lambda q}{4\pi}\right)^2}\right)^2 - 1\right)\Delta I_{\mathrm{aniso}}(q) + \Delta I_{\mathrm{iso}}(q). \quad (1)$$

The analysis of anisotropic scattering signal has been discussed in detail previously[2]. In short, the anisotropic component of the signal is caused by the preferential excitation of those molecules whose transition dipole moments (TDM) are oriented parallel to the linear polarization of the optical pump pulse in the laboratory frame. Because of the quadrant symmetry of the $\cos^2(\theta)$ distribution in the ensemble for a single-photon excitation process, this angular symmetry can be described by second order Legendre polynomials. The total scattering pattern $\Delta I(\phi, q)$ thus can be decomposed to an isotropic component $\Delta I_{\mathrm{iso}}(q)$ that reflects all the intrinsic information of the molecule in the molecular frame and an anisotropic component $\Delta I_{\mathrm{aniso}}(q)$ that reflects additional information of the rotational motions of the molecule in the laboratory frame.

Supplementary Note 2. Analysis of Pump-Probe Scattering Signal

During the experiment, time zero and the cross correlation of the pump and probe pulses were estimated from the rise of the UV-pulse induced signal. The time dependent scattering signals integrated in two specific q regions (0.3-1.6 Å and 1.7-2.5 Å, respectively) and the fit results are shown in Supplementary Figure 1. Supplementary Equation 2 for $f(t)$, convoluted with a Gaussian instrument function $g(t)$, is used for modeling the time dependent experimental signals.

$$f(t) = H(t > t_0) \cdot \left[Ae^{-\frac{t-t_0}{\tau}} + B\left(1 - e^{-\frac{t-t_0}{\tau}}\right) + C\right]$$

$$g(t) = \frac{1}{\sigma\sqrt{2\pi}} e^{-\frac{1}{2}\left(\frac{t-t_0}{\sigma}\right)^2} \quad (2)$$

The $g(t)$ describes the time correlation between the optical pump pulse and the x-ray probe pulse, where t_0 is the time of UV excitation, and σ is the Gaussian width. The $f(t)$ models electronic and nuclear process in the molecule after initial excitation, where τ is the time

constant of the *3p* relaxation process. Specifically, the initial onset of the signal from the initially prepared *3p* state induced by the optical excitation is characterized by a Heaviside step-function $H(t > t_0)$, the subsequent decay of this onset signal originating from the relaxation of the initially prepared 3p state is represented by an exponential function $Ae^{-\frac{t-t_0}{\tau}}$, and the simultaneous rise of a new signal originating from the appearance of relaxed ground state hot CHD molecules during same relaxation process is represented by the exponential function $B\left(1 - e^{-\frac{t-t_0}{\tau}}\right)$. The constant *C* accounts for the baseline of the background noise.

The time dependencies of the two q regions were fitted simultaneously with shared parameters (t_0, σ, τ and *C*). Because the scattering pattern in the two q regions respond differently to the changes in the molecule, *A* and *B* are kept as independent parameters in the two fits. As shown in the main text, because the near instantaneous change in electronic structure upon excitation mainly affects the low q region (0.3-1.6 Å, shown in black in Supplementary Figure 1) of the percent difference scattering signal rather than the high q region (1.7-2.5 Å, shown in red in Supplementary Figure 1), the value of *A* determined from the fit in the low q region (*A* = 2.124) is much larger than the value determined from the fit in the high q region (*A* = 0.002). The shared parameters are determined to be t_0 = 31 (-16 ~ 79) fs, σ = 117 (67 ~ 167) fs, τ = 201 (84 ~ 318) fs, with 95% confidence intervals given in the parentheses.

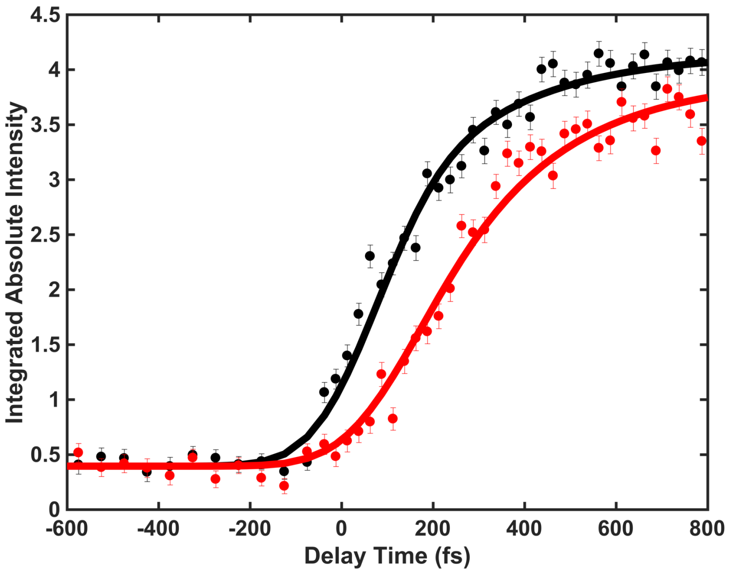

Supplementary Figure 1. Analysis of time-dependent scattering signals. The experimental time-dependent scattering signals are integrated in two separate q regions: low q (0.3-1.6 Å) shown as black dots, and high q (1.7-2.5 Å) shown as red dots. The estimated shot noise errors are shown with 1σ error bars. The resulting fits are shown as black and red curves.

Supplementary Note 3. Radial Distribution Function

The difference radial distribution function for the 25 fs time bin is obtained from the fraction signal difference discussed in the main text via a multiplication by the theoretical *ab-initio* signal of the unpumped system, $I_{\text{off}}^{\text{th}}(q)$. This gives direct access to the quantity $\Delta I(t,q) = I_{\text{on}}(t,q) - I_{\text{off}}(q)$. This procedure is considered optimal for the reduction of noise and background fluctuations as compared to starting from the raw $I_{\text{on}}(t,q)$ and $I_{\text{off}}(q)$.[3,4] The difference signal, $\Delta I(t,q)$, is then fitted with 9*th* degree polynomial to minimize noise and provide high resolution in q space, which the sine-transform requires for numerical stability. The difference radial distribution function is then obtained from:

$$\Delta\text{RDF}(r) = 2/\pi \int_0^\infty qr[I_{\text{on}}(q) - I_{\text{off}}(q)]\sin(qr)\, W(q)\, dq, \qquad (3)$$

where $W(q)$ is the modified Gaussian window function:

$$W(q) = \begin{cases} 1, & q < 1.8\ \text{Å}^{-1} \\ \exp\{-0.7(q-1.8)^2\}, & q \geq 1.8\ \text{Å}^{-1} \end{cases}. \qquad (4)$$

$W(q)$ is used to reduce the numerical artefacts of the transform associated with the finite width of the q range. The limited q range also results in relatively low resolution in real space of ~ 0.75 Å, which follows from the uncertainty relationship $\sigma_q \sigma_r \sim \pi$. The effect of the resolution can be observed in Supplementary Figure 2, which compares the theoretical and the experimental $\Delta \mathrm{RDF}(r)$. The theoretical difference radial distribution function is obtained from a sine-transform of the *ab-initio* scattering data, $I_{\mathrm{exc}}(q, \mathbf{R}') - I_{\mathrm{X}}(q, \mathbf{R}_0)$, which can be further separated into an electronic part, $I_{\mathrm{exc}}(q, \mathbf{R}') - I_{\mathrm{X}}(q, \mathbf{R}')$, and the nuclear part, $I_{\mathrm{X}}(q, \mathbf{R}') - I_{\mathrm{X}}(q, \mathbf{R}_0)$. The q range of the theoretical scattering signal is 0-50 $Å^{-1}$ and, hence, no window function is needed. While the experimental difference radial distribution function clearly follows the theoretical profile, the details of nuclear rearrangements associated with the range < 5 Å are lost. However, the distribution shows clearly the increase in electron density at large distances associated with the excitation to a diffuse Rydberg electronic state, and the corresponding decrease in small r (otherwise modulated by nuclear rearrangement region below 5 Å). It should be pointed out that, on account of the average C-C bond distances and angle constrains, no molecular geometry (nuclear configuration), *e.g.* corresponding to the ring-opened molecule, can give raise to changes in the distribution above approximately $r > 5.0$ Å, and that this signal thus must reflect changes due to the diffuse nature of the excited electronic state.

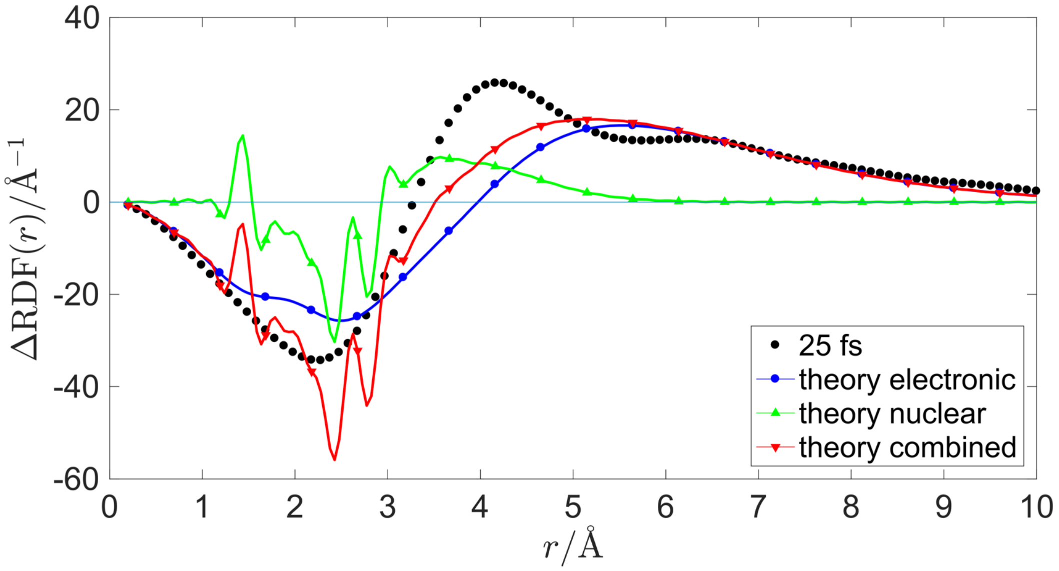

Supplementary Figure 2. Experimental and theoretical real-space difference radial distribution functions. The experimental curve is obtained from the experimental data at 25 fs pump-probe delay time. The theoretical ΔRDF*(r)* is a sum of two contributions, nuclear and electronic, in analogy with the fractional difference signal presented in the main text. The theoretical curves are obtained from a sine-transform of the theoretical scattering patterns.

Supplementary Note 4. Thermal Structural Distribution Due to Nuclear Vibrations

Because the electronic contribution to the scattering signal is comparatively constant with regards to molecular geometry, as we have shown in Figure 3 (a) in the main text, the confidence interval for the calculated scattering signal of the 3p electronic state is mainly due to the distribution of molecular geometries near the 3p minimum energy structure. In the following we model this as a thermal distribution. Based on previous experimental and computational results, we know that the excitation energy of 3p state is about 6.05 eV (see Supplementary Table 1). At 200 nm UV excitation (6.2 eV photon energy), the difference between photon energy and the 3p excitation energy is thus about 0.15 eV. The excess kinetic energy that can be redistributed to the vibrational degrees of freedom in the molecule is thus small, and the molecule in the 3p state is comparatively cold (~470 K, assuming harmonic vibrations).

To model the vibrational distribution of CHD properly, we calculate the vibrational normal modes of CHD at the ion minimum-energy structure, which is a good approximation of

the 3p minimum-energy structure, using *ab-initio* electronic structure calculations at the CASPT2(3,4)/aug-cc-pVDZ level of theory in the software package Molpro. An ensemble of 10,000 geometries is sampled from the quantum Wigner distribution at 470 K based on the calculated vibrational normal modes. Each of the 10,000 geometries is used to simulate a scattering pattern using the independent atom model (IAM), as IAM remains a valid approximation for the nuclear component of the scattering. Finally, the standard deviation, *σ(q)*, of the simulated scattering patterns for the 10,000 sampled structures is calculated, and this is used to estimate the thermal structural distribution (effect of molecular geometry distribution) for the calculated 3p signal, presented as the shaded area in Figure 2 (b) of the main text.

Supplementary Note 5. Derivation of Theoretical Fractional Difference

The fractional difference scattering signal $\Delta S_{\mathrm{exc}}(q,t)$ of a molecule at pump-probe delay time t is defined as,

$$\Delta S_{\mathrm{exc}}(q,t) = \frac{I_{\mathrm{on}}(q,t) - I_{\mathrm{off}}(q,t_0)}{I_{\mathrm{off}}(q,t_0)}, \tag{5}$$

where $I_{\mathrm{on}}(q,t)$ is the scattering signal measured at the given pump-probe delay time after the molecule has been excited by the pump pulse (pump pulse on) and $I_{\mathrm{off}}(q,t_0)$ refers to the scattering signal prior to excitation (pump pulse off). If the molecule is initially in its electronic ground state X, the pump-off signal can be written as the static scattering signal of the molecule in its electronic ground state, *i.e.*,

$$I_{\mathrm{off}}(q,t_0) = I_{\mathrm{X}}(q,t_0). \tag{6}$$

Since the experiment is set up to avoid multiphoton processes, we need not consider secondary ground state wave packets and the pump-on signal will have two components. The first corresponds to the fraction excited by the pump, given by γ, and the second to the $1-\gamma$ fraction that is unaffected by the pump. The $I_{\mathrm{on}}(q,t)$ signal can then be written as,

$$I_{\mathrm{on}}(q,t) = \gamma \cdot I_{\mathrm{exc}}(q,t) + (1-\gamma) \cdot I_{\mathrm{X}}(q,t_0), \tag{7}$$

where $I_{\mathrm{exc}}(q,t)$ is the scattering signal from the excited component. It is worth noting that at longer pump-probe delay times than those considered here, $I_{\mathrm{exc}}(q,t)$ may decay to the electronic ground state as the molecule undergoes internal conversion. Inserting the definitions in Supplementary Equations (6) and (7) into the Supplementary equation (5) yields,

$$\Delta S_{\mathrm{exc}}(q,t) = \gamma \cdot \frac{I_{\mathrm{exc}}(q,t) - I_{\mathrm{X}}(q,t_0)}{I_{\mathrm{X}}(q,t_0)}. \tag{8}$$

If we disregard γ as a simple multiplicative scaling factor and express the scattering signal as a function of time-dependent classical nuclear structures instead of the pump-probe delay time t itself, Supplementary Equation (8) simplifies to,

$$\Delta S_{\mathrm{exc}}(q,\mathbf{R}') = \frac{I_{\mathrm{exc}}(q,\mathbf{R}') - I_{\mathrm{X}}(q,\mathbf{R}_0)}{I_{\mathrm{X}}(q,\mathbf{R}_0)}. \tag{9}$$

where $\mathbf{R}'$ is the nuclear geometry of the molecule on the excited state at pump-probe delay t and $\mathbf{R}_0$ refers to the equilibrium structure of the molecule on the electronic ground state at t_0 prior to the excitation. Supplementary Equation (9) corresponds to Equation (3) in the main body of the letter.

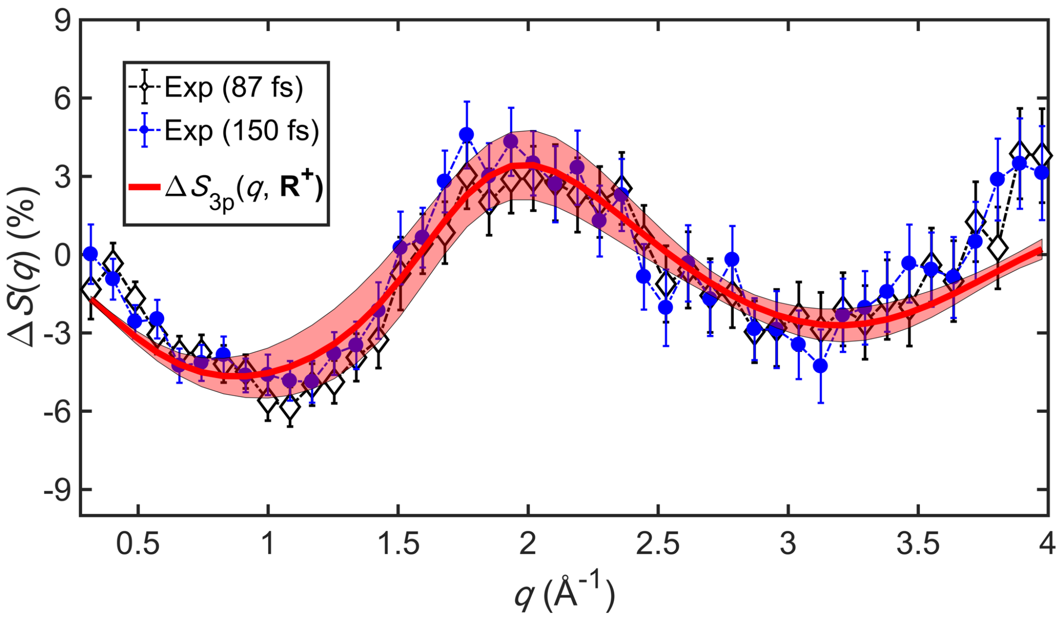

Supplementary Figure 3. Experimental and theoretical fractional difference signals, $\boldsymbol{\Delta S(q)}$, shown in percent. The experimental signal at 87 fs and 150 fs delay time are shown in black diamonds and blue circles with 1σ error bars. Both curves have been deconvoluted with an instrument function and divided by the excitation fraction. The theoretical $\Delta S_{3\mathrm{p}}(q, \mathbf{R}^{+})$ signal for the electronic 3p state is shown in red with the shaded region accounting for the sampling of geometries in the excited state.

	CASSCF[5] /eV	CASPT2[5] /eV	Experiment[5] /eV	CASPT2/eV (This work)
1B	8.40	4.72	4.94	4.72
3s	5.83	5.49	5.39	5.39
$3p_x$	6.43	5.98	6.03	6.05
$3p_y$	6.50	6.04	--------------	5.97
$3p_z$	6.36	6.12	6.05	6.06

Supplementary Table 1. Excitation energies for the first five low-lying excited states of CHD. First three columns: calculated results using Complete Active Space Self-Consistent Field theory (CASSCF), second-order Complete Active Space Perturbation Theory (CASPT2) and experimental results reported in Table 4 of Supplementary Reference 5. CASPT2 (this work): calculated results using the method applied for the present study, details of the CASPT2 calculation could be found in the Methods section.

Supplementary References

1 Lorenz U., Møller K. B. & Henriksen N. E. On the interpretation of time-resolved anisotropic diffraction patterns. *New J. Phys.*, **12**, 113022 (2010).

2 Yong, H. *et al*. Determining orientations of optical transition dipole moments using ultrafast x-ray scattering. *J. Phys. Chem. Lett.*, **9**, 6556-6562 (2018).

3 Budarz, J. M. *et al.* Observation of femtosecond molecular dynamics via pump-probe gas phase x-ray scattering. *J. Phys. B: At. Mol. Opt. Phys.*, **49**, 034001 (2016).

4 Ruddock, J. M. *et al.* Simplicity beneath complexity: counting molecular electrons reveals transients and kinetics of photodissociation reactions, *Angew. Chem. Int. Ed.*, **58**, 6371-6375 (2019).

5 Merchán M. *et al.* Electronic Spectra of 1,4-cyclohexadiene and 1,3-cyclohexadiene: a combined experimental and theoretical investigation. *J. Phys. Chem. A*, **103**, 5468-5476 (1999).
